# Comparative appraisal of mono and hybrid nanofluid flows comprising carbon nanotubes over a three-dimensional surface impacted by Cattaneo–Christov heat flux

**DOI:** 10.1038/s41598-023-34686-8

**Published:** 2023-05-17

**Authors:** Khalid Abdulkhaliq M. Alharbi, Muhammad Ramzan, Nazia Shahmir, Hassan Ali S. Ghazwani, Yasser Elmasry, Sayed M. Eldin, Muhammad Bilal

**Affiliations:** 1 Mechanical Engineering Department, College of Engineering, Umm Al-Qura University, 24382 Mecca, Kingdom of Saudi Arabia; 2 Department of Computer Science, Bahria University, Islamabad, 44000 Pakistan; 3 Department of Mechanical Engineering, Faculty of Engineering, Jazan University, 45124 Jazan, Kingdom of Saudi Arabia; 4 Department of Mathematics, College of Sciences, King Khalid University, 61413 Abha, Saudi Arabia; 5 Department of Mathematics, Faculty of Science, Al-Azhar University, Assiut, Egypt; 6 Center of Research, Faculty of Engineering, Future University in Egypt, New Cairo, 11835 Egypt; 7 Department of Mathematics, University of Chenab, Gujrat, 50700 Pakistan

**Keywords:** Mechanical engineering, Software

## Abstract

Carbon nanotubes (CNTs) are nanoscale tubes made of carbon atoms with unique mechanical, electrical, and thermal properties. They have a variety of promising applications in electronics, energy storage, and composite materials and are found as single-wall carbon nanotubes (SWCNTs) and double-wall carbon nanotubes (DWCNTs). Considering such alluring attributes of nanotubes, the motive of the presented flow model is to compare the thermal performance of magnetohydrodynamic (MHD) mono (SWCNTs)/Ethylene glycol) and hybrid (DWCNTs- SWCNTs/Ethylene glycol) nanofluids over a bidirectional stretching surface. The thermal efficiency of the proposed model is gauged while considering the effects of Cattaneo-Christov heat flux with prescribed heat flux (PHF) and prescribed surface temperature (PST). The flow is assisted by the anisotropic slip at the boundary of the surface. The system of partial differential equations (PDEs) is converted into a nonlinear ordinary differential system by the use of similarity transformations and handled using the bvp4c numerical technique. To depict the relationship between the profiles and the parameters, graphs, and tables are illustrated. The significant outcome revealed that the fluid temperature rises in the scenario of both PST and PHF cases. In addition, the heat transfer efficiency of the hybrid nanoliquid is far ahead of the nanofluid flow. The truthfulness of the envisioned model in the limiting scenario is also given.

## Introduction

Nanofluids are suspensions of nanoparticles in a base liquid. The nanoparticles can be made of metals, oxides, or other materials, and they can enhance the thermal conductivity and/or the heat capability of the fluid base. This makes nanofluids particularly useful for applications such as cooling and heating, where their enhanced heat transfer properties can improve system performance. The heat transfer rate of nanoliquids is greater compared to the same volume of base fluid (without nanoparticles) due to their augmented thermal conductivity and heat capacity. The insertion of nanoparticles, which provide additional thermal routes for heat transmission, improves heat transfer in nanofluids. The exact enhancement of heat transfer relies on several factors, such as the type and size of nanoparticles, the volume fraction of nanoparticles in the liquid, the thermal properties of the liquid and nanoparticles, and the flow conditions. Carbon nanotubes (CNTs) play a significant role in nanofluids due to their unique physical and thermal properties. CNTs are cylindrical structures made of graphene sheets, and they have high thermal conductivity, a high aspect ratio, and a large surface area. These properties make CNTs ideal for use as nanoparticles in nanofluids, where one can achieve the overall heat transfer performance of the fluid. In addition to their thermal properties, CNTs also have high mechanical strength, low density, and high electrical conductivity, which makes them useful for a variety of applications beyond heat transfer. The use of CNTs in nanofluids is still an active area of research, and further explorations are required to fully understand the function of CNT-based nanofluids and to optimize their use for specific applications. Chougule and Sahu^1^ analyzed the thermal performance of an automobile’s radiator comprising a nanofluid with CNTs and a water mixture. The salient conclusion revealed that nano coolant has an edge over water while discussing the rate of mass flux. The thermal performance of the CNTs-based liquid (water) in a flat heated pipe is studied by Arya et al.^2^ The important outcomes revealed that the heat transfer rate triggers the evaporating process. It is also witnessed that mass concentration also increases when the heat transfer coefficient is boosted. The assesment of the hybrid nanofluid on a cylinder and plane sheet considering the Yamada–Ota hybrid nanofluid model is deliberated numerically by Ramzan et al.^3^ using Keller box approximation. The highlighted outcome is that the flow velocity, as well as temperature, depict opposing behavior when influenced by a strong magnetic field. Farooq et al.^4^ computed the hybrid nanofluid flow comprising CNTs/ethylene glycol over an elongated surface supported by irreversibility analysis. Entropy generation for the flow velocity component is diminishing. The flow of the hybrid nanofluid containing combined effects of the ferro-oxide/CNTs nanomaterials and water between two parallel horizontally placed plates impacted by a magnetic field is numerically studied by Qureshi et al.^5^ The heat transfer rate of the upper plate is increasing for a thermal radiation value greater than zero. Nabi et al.^6^ analyzed numerically the nanofluid flow models comprising (SWCNTs-Water) and (MWCNTs-Water) through a micro-channel using the Finite Volume method. The outcomes disclosed that equal distribution of the fluid in the microchannel results in the highest heat transfer rate. Some recent explorations discussing the role of CNTs in nano/hybrid nanofluid flows may be found in^7–10^.

The Cattaneo-Christov heat flux is an extended form of the Fourier heat flux equation that considers the effects of heat conduction and thermal diffusion. It is a non-Fourier type heat transfer equation, which means that it provides a more accurate description of heat transfer in materials with high thermal diffusion rates, such as high-conductivity materials. The Cattaneo-Christov heat flux equation contributes a major role in the performance of the heat transfer in the nanoliquid flows. Nanofluids are suspensions of nanoparticles in a fluid, and their behavior is influenced by several factors, including the presence of nanoparticles, which can significantly affect the thermal conductivity and heat generation within the fluid. The Cattaneo-Christov heat flux equation takes thermal diffusion into account, which can be a significant issue in nanofluid flows. Furthermore, the Cattaneo-Christov heat flux equation can be used to analyse thermal instability in nanofluid flows, which can occur due to the enhance thermal conductivity of the nanoparticles and the fluid's thermal diffusion. This information can be utilized to improve the design of nanofluid-based cooling and energy generation systems. Studies have shown that the Cattaneo-Christov heat flux term can have a significant consequence on the heat transfer attributes of nanofluids, especially in laminar flow regimes. However, the applicability of the Cattaneo-Christov heat flux term in nanofluid flows is still a topic of ongoing research, as its effectiveness can be influenced by varied variables such as particle concentration, size and type, fluid properties, and flow conditions. Gowda et al.^11^ numerically computed the non-Newtonian nanofluid three-dimensional flow influenced by a magnetic field and Cattaneo-Christov heat flux. Two different flow combinations namely Nanolubricant, and Propylene-Water mixture and Paraffin wax flow are considered. It is witnessed that nano lubricant flow depicts a escalating rate of heat transfer than the propylene-water mixture and Paraffin wax flow. The flow of hybrid nanofluid comprising silver-copper oxide/kerosene oil past a bi-directional stretched surface impacted by Cattaneo-Christov double diffusion and Hall current is studied by ZeinEldin et al.^12^ The outcome inferred that the boundary layer thickness is on the decline for large values of the Prandtl number. Shah et al.^13^ using the shooting technique deliberated the Prandtl-Eyring hybrid nanofluid flow with thermal radiative flux amalgamated with modified fourier law in a permeable media. The important results revealed that the liquid velocity substantially decelerates for the upsurging estimations of the permeable factor. The impact of the melting heat and Cattaneo-Christov heat flux on a Casson hybrid Ferrohydrodynamic nanofluid (Fe_3_O_4_–Ag/blood) flow over an elongated surface accompanying irreversibility analysis is analyzed by Jakeer et al.^14^ It is comprehended in this exploration that hybrid nanofluid temperature is decreased for the ferromagnetic parameter. Hayat et al.^15^ numerically computed the flow the hybrid nanoliquid flow in a channel due to Cattaneo-Christov heat flux in a Darcy-Forchheimer spongy medium. The upper plate is moving to and fro whereas the convective condition is implied on the lower one. Irreversibility assessment is also executed in this work. It is interpreted that the large estimations of the squeezing parameter influence the Entropy generation rate. Some more recent investigations depict the consequence of Cattaneo-Christov heat flux on varied geometries^16–23^.

Boundary layer flows over extended surfaces attracted researchers owing to their wide-ranging applications like hot rolling, wire drawing, elastic sheets, crustal growing, etc. In the case of stretched surfaces, the role of effective cooling is vital in obtaining the refined end product. Wang^24^ analytically discussed the flow of viscous fluid generated by the two-dimensional extended flat sheet. In this study, it is comprehended that for axisymmetric stretching (stretching in the same directions), the ratio of the stretching rate is equal to one. The study was corroborated by comparing it with Crane^25^ in a limiting case when the stretching ratio is zero. Later, Liu and Andersson^26^ investigated the bidirectional stretching sheet impact on the constant three-dimensional viscous fluid flow. The results revealed that fluid temperature declined for higher estimates of the stretching ratio parameter. Joshi et al.^27^ illustrated the hybrid nanofluid flow along the bidirectional spongy surface with the existence of heat generation. Ramzan and Yousaf^28^ explored the steady flow of viscoelastic nanoliquid over an extending surface. It is noticed that both the velocities exhibit opposing behavior when evaluated versus the stretching parameter. Interestingly, an anisotropic slip which is a directional-dependent slip can also be employed on bidirectional stretching surfaces. According to Wang^29^, anisotropic slip in fluid flow indicates that the slip factor varies with the flow direction. Amirsom et al.^30^ explored the three-dimensional flow of nanofluids over a bidirectional stretched sheet with an anisotropic slip and gyrotactic microorganisms. References^31–33^ are given about the bidirectional stretching surface in numerous scenarios.

Prescribed surface temperature (PST) and prescribed heat flux (PHF) are two types of boundary conditions used in numerical simulations of heat transfer. In the condition of the prescribed surface temperature, the temperature at a specific surface is set to a particular value, which is kept constant throughout the simulation. The temperature at the boundary is identified as a boundary condition. Nevertheless, considering the case of prescribed heat flux, the heat transfer rate (heat flux) at a particular surface is set to a specific value. This type of boundary condition is used when the heat transfer rate is established or controlled, and it is required to establish the resulting temperature. Both PST and PHF temperatures can be used in numerous applications, such as electronics cooling, energy systems, and thermal insulation design. In the case of nanofluids, these boundary conditions are crucial for understanding the heat transfer characteristics of the fluid, including its thermal conductivity, convective heat transfer, and temperature distribution. By using these boundary conditions, researchers and engineers can develop more efficient heat transfer systems, improve energy efficiency, and optimize thermal management solutions in various applications. A plethora of publications may be quoted that highlight the importance of both types of boundary conditions. Chandel and Sood^34^ investigated the effects of the PST and the PHF heating constraints on the unsteady flow of a Williamson nanofluid across an extendable surface embedded in a spongey medium. It is found that under both temperature conditions (PST/PHF), the heat transfer rate increases which may be useful in cooling procedures. Ahmad et al.^35^ numerically analyzed the flow of the hybrid nanoliquid with prescribed thermal conditions and thermal radiative heat flux using the Keller Box technique. It is determined that the liquid temperature is maximum for the thermal conditions. The flow of the Maxwell nanofluid flow with the impact of Arrhenius energy and prescribed temperature conditions over an extended bi-directional surface is studied by Faisal et al.^36^ It is witnessed that the liquid temperature is higher in the case of the prescribed heat flux than the prescribed surface temperature. Some more investigations featuring PST and PHF may also be seen in^37–39^.

The aforementioned cited studies revealed that studies focusing on the isothermal temperature may be found in bulk. But lesser attention is paid to the nanofluid flows with PST and PHF temperatures. This channel even becomes narrower when nanofluid flow over a bidirectional extended sheet is discussed. However, no attempt is made so far that deliberated the comparison of mono/hybrid nanofluids due to a bi-directionally stretched sheet with anisotropic slip and prescribed temperatures, *i.e.,* PST and PHF. The inimitability of the current model is strengthened with the inclusion of the Cattaneo-Christov heat flux to study the heat equation. The fundamental nonlinear partial differential equations (PDEs) that represent energy and momentum are converted into ODEs and numerically computed. Figures and tables are employed to determine the findings.

## Mathematical analysis

**Figure 1:**
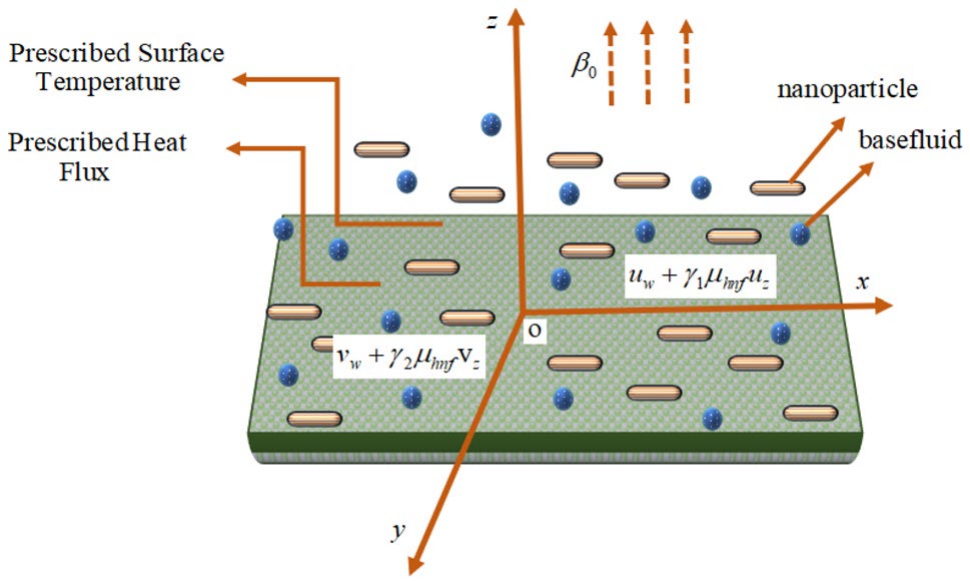
Schematic of the flow pattern.

Consider the laminar, steady, incompressible flows of mono and hybrid nanofluids due to a bidirectional extended sheet with anisotropic slip. The plate is placed in the $xy -$ plane at $z = 0$. Let $u_{w}$ and $v_{w}$ be the stretching plate velocities along with two lateral directions. A magnetic field is employed normally to the surface along $z -$ axis with magnetic field strength $\beta_{0} .$ Prescribed surface temperature and prescribed heat flux are two thermal conditions that are imposed on the surface. The power indices $r^{ * }$ and $s^{ * }$ in the thermal boundary conditions illustrate the temperature and the heat flux at the wall changes in the $x$ and $y$ plane. Figure 1 depicts the flow model as well as the physical coordinate system. Table 1 demonstrates the thermal and physical features of working fluid and immersed nanoparticles.

**Table1:** Physical and thermal properties of ethylene glycol (working fluid), SWCNTs, and DWCNTs (nanoparticles)^40–42^:

Working fluid/nanoparticles	$\left( \rho \right)$	$\left( {C_{p} } \right)$	$\left( k \right)$	$\left( \sigma \right)$
DWCNT	2100	710	3000	$1 \times 10^{6}$
Ethylene Glycol	1109	2382	2.84	0.01485
SWCNT	2100	600	3500	$1 \times 10^{ - 12}$

Ethylene glycol and SWCNTs/DWCNTs are postulated to be in thermal equilibrium with no interference between them. Following these considerations and the Tiwari and Das^35^ nanofluid model, we have:

1 $$\frac{\partial u}{{\partial x}} + \frac{\partial v}{{\partial y}} + \frac{\partial w}{{\partial z}} = 0,$$

2 $$u\frac{\partial u}{{\partial x}} + v\frac{\partial u}{{\partial y}} + w\frac{\partial u}{{\partial z}} = \nu_{hnf} \left( {\frac{{\partial^{2} u}}{{\partial x^{2} }} + \frac{{\partial^{2} u}}{{\partial y^{2} }} + \frac{{\partial^{2} u}}{{\partial z^{2} }}} \right) - \left( {\frac{{\sigma_{hnf} \beta_{o}^{2} }}{{\rho_{hnf} }}} \right)u,$$

3 $$u\frac{\partial v}{{\partial x}} + v\frac{\partial v}{{\partial y}} + w\frac{\partial v}{{\partial z}} = \nu_{hnf} \left( {\frac{{\partial^{2} v}}{{\partial x^{2} }} + \frac{{\partial^{2} v}}{{\partial y^{2} }} + \frac{{\partial^{2} v}}{{\partial z^{2} }}} \right) - \left( {\frac{{\sigma_{hnf} \beta_{o}^{2} }}{{\rho_{hnf} }}} \right)v,$$

4 $$u\frac{\partial T}{{\partial x}} + v\frac{\partial T}{{\partial y}} + w\frac{\partial T}{{\partial z}} + \lambda \left[ \begin{gathered} \left( {u\frac{\partial u}{{\partial x}} + v\frac{\partial u}{{\partial y}} + w\frac{\partial u}{{\partial z}}} \right)\frac{\partial T}{{\partial x}} + u^{2} \frac{{\partial^{2} T}}{{\partial x^{2} }} + v^{2} \frac{{\partial^{2} T}}{{\partial y^{2} }} + w^{2} \frac{{\partial^{2} T}}{{\partial z^{2} }} + \hfill \\ \left( {u\frac{\partial v}{{\partial x}} + v\frac{\partial v}{{\partial y}} + w\frac{\partial v}{{\partial z}}} \right)\frac{\partial T}{{\partial y}} + 2uw\frac{{\partial^{2} T}}{\partial x\partial z} + 2uv\frac{{\partial^{2} T}}{\partial x\partial y} \hfill \\ 2vw\frac{{\partial^{2} T}}{\partial y\partial z} + \left( {u\frac{\partial w}{{\partial x}} + v\frac{\partial w}{{\partial y}} + w\frac{\partial w}{{\partial z}}} \right)\frac{\partial T}{{\partial z}} \hfill \\ \end{gathered} \right] = \frac{{k_{hnf} }}{{\left( {\rho C_{p} } \right)_{hnf} }}\left( {\frac{{\partial^{2} T}}{{\partial z^{2} }}} \right).$$

With applicable boundary conditions^20^:

5 $$\begin{gathered} u\left( 0 \right) = u_{w} + \gamma_{1} \mu_{hnf} \frac{\partial u}{{\partial z}},\,\,\,\,\,\,u\left( \infty \right) \to 0,\,\,\,\,\,v\left( 0 \right) = v_{w} + \gamma_{2} \mu_{hnf} \frac{\partial v}{{\partial z}},\,\,\,\,\,\,w\left( 0 \right) = 0,\,\,\,\,\,v\left( \infty \right) \to 0, \hfill \\ \left. \begin{gathered} PST\,\,T\left( 0 \right) = T_{w} ,\,\,\,\,\,\,\,\,\,\,\,\,\,\,\,T\left( \infty \right) \to T_{\infty } \, \hfill \\ PHF\,\,\frac{\partial T}{{\partial z}} = - \left. {\frac{{q_{w} }}{{k_{hnf} }}} \right|_{{\left( {\eta = 0} \right)}} ,\,\,\,T\left( \infty \right) \to T_{\infty } \hfill \\ \end{gathered} \right\}, \hfill \\ \end{gathered}$$

where in Eq. (5), $q_{w} = T_{1} x^{{r^{ * } }} y^{{s^{ * } }} .$ Further, the non-dimensional similarity transformations are listed below:

6 $$\begin{aligned} \eta & = z\sqrt {\frac{a}{{\nu_{f} }}} ,\,\,\,\,u = axf^{^{\prime}} \left( \eta \right),\,\,\,\,v = ayg^{^{\prime}} \left( \eta \right),\,\,\,\,\,w = - \sqrt {a\nu_{f} } \left( {f\left( \eta \right) + g\left( \eta \right)} \right), \\ PST:\,\,\,\theta \left( \eta \right) & = \frac{{T\left( {x,y,z} \right) - T_{\infty } }}{{T_{w} \left( {x,y} \right) - T_{\infty } }},\,\,\,\,\,\,\,where\,\,T\left( {x,y,z} \right) = T_{o} x^{{r^{ * } }} y^{{s^{ * } }} + T_{\infty } = T_{w} \left( {x,y} \right), \\ PHF:\,\,\,\Theta \left( \eta \right) & = \frac{{T\left( {x,y,z} \right) - T_{\infty } }}{{T_{w} \left( {x,y} \right) - T_{\infty } }},\,\,\,\,\,where\,\,T\left( {x,y,z} \right) = \frac{{T_{1} }}{{k_{hnf} }}\sqrt {\frac{{\nu_{f} }}{a}} x^{{r^{ * } }} y^{{s^{ * } }} + T_{\infty } = T_{w} \left( {x,y} \right), \\ \end{aligned}$$

Under the influence of preceding transformations, Eq. (1) is satisfied, and the results of Eqs. (2), (3), and (4) are as follows:

7 $$\left( {\frac{{\nu_{hnf} }}{{\nu_{f} }}} \right)f^{^{\prime\prime\prime}} \left( \eta \right) + \left( {f\left( \eta \right) + g\left( \eta \right)} \right)f^{^{\prime\prime}} \left( \eta \right) - f^{^{\prime}2} \left( \eta \right) - \left( {\frac{{\sigma_{hnf} }}{{\sigma_{f} }}} \right)\left( {\frac{{\rho_{f} }}{{\rho_{hnf} }}} \right)M_{n} f^{^{\prime}} \left( \eta \right) = 0,$$

8 $$\left( {\frac{{\nu_{hnf} }}{{\nu_{f} }}} \right)g^{^{\prime\prime\prime}} \left( \eta \right) + \left( {f\left( \eta \right) + g\left( \eta \right)} \right)g^{^{\prime\prime}} \left( \eta \right) - g^{^{\prime}2} \left( \eta \right) - \left( {\frac{{\sigma_{hnf} }}{{\sigma_{f} }}} \right)\left( {\frac{{\rho_{f} }}{{\rho_{hnf} }}} \right)M_{n} g^{^{\prime}} \left( \eta \right) = 0,$$

9 $$\begin{gathered} \left( {\frac{{k_{hnf} }}{{k_{f} }}} \right)\left( {\frac{{\left( {\rho C_{p} } \right)_{f} }}{{\left( {\rho C_{p} } \right)_{hnf} }}} \right)\theta^{^{\prime\prime}} \left( \eta \right) + {\text{Pr}}\left( {f\left( \eta \right) + g\left( \eta \right)} \right)\theta^{\prime}\left( \eta \right)\, - {\text{Pr}}\left( {r^{ * } f^{^{\prime}} \left( \eta \right) + s^{ * } g^{^{\prime}} \left( \eta \right)} \right)\theta \left( \eta \right)\, - \hfill \\ \Pr \lambda_{cc} \left[ \begin{gathered} r^{ * } \left( {r^{ * } - 1} \right)\theta f^{^{\prime}2} + s^{ * } \left( {s^{ * } - 1} \right)\theta g^{^{\prime}2} - \left( {f + g} \right)^{2} \theta^{^{\prime\prime}} + 2r^{ * } s^{ * } f^{^{\prime}} g^{^{\prime}} \theta - 2r^{ * } s^{ * } \left( {f + g} \right)\theta^{^{\prime}} f^{^{\prime}} \hfill \\ - 2s^{ * } \left( {f + g} \right)g^{^{\prime}} \theta^{^{\prime}} + \left( {s^{ * } g^{^{\prime}2} + r^{ * } f^{^{\prime}2} } \right)\theta - \left( {f + g} \right)\theta \left( {r^{ * } f^{^{\prime\prime}} + s^{ * } g^{^{\prime\prime}} } \right) + \left( {f + g} \right)\left( {f^{^{\prime}} + g^{^{\prime}} } \right)\theta^{^{\prime}} \left( \eta \right) \hfill \\ \end{gathered} \right] = 0. \hfill \\ \end{gathered}$$

The transformation of boundary conditions is as follows:

10 $$\begin{gathered} f^{^{\prime}} \left( 0 \right) = 1 + \Gamma_{1} \frac{{\mu_{hnf} }}{{\mu_{f} }}f^{^{\prime\prime}} \left( 0 \right),\;\;f\left( 0 \right) = 0,\;\;g^{^{\prime}} \left( 0 \right) = \alpha + \Gamma_{2} \frac{{\mu_{hnf} }}{{\mu_{f} }}g^{^{\prime\prime}} \left( 0 \right),\;\;g\left( 0 \right) = 0,\;\;f^{^{\prime}} \left( \infty \right) = 0,\;\;g^{^{\prime}} \left( \infty \right) = 0, \hfill \\ \left. \begin{gathered} PST:\,\,\,\,\,\theta \left( 0 \right) = 1;\,\,\,\,\,\,\,\,\,\theta \left( \infty \right) = 0 \hfill \\ PHF:\,\,\,\Theta^{\prime}\left( 0 \right) = - 1;\,\,\,\Theta \left( \infty \right) = 0\,\, \hfill \\ \end{gathered} \right\}. \hfill \\ \end{gathered}$$

### Thermal and physical properties of mono/hybrid nanofluid

Mono/hybrid nanofluids' effective dynamic viscosity is formulated as:

11 $$\frac{{\mu_{nf} }}{{\mu_{f} }} = \frac{1}{{\left( {1 - \varphi_{SWCNT} } \right)^{2.5} }},\;\;\;\frac{{\mu_{hnf} }}{{\mu_{f} }} = \frac{1}{{\left( {1 - \varphi_{SWCNT} - \varphi_{DWCNT} } \right)^{2.5} }}.$$

Mono/hybrid nanofluids' effective density is expressed as:

12 $$\begin{aligned} \frac{{\rho_{nf} }}{{\rho_{f} }} & = \left( {1 - \varphi_{SWCNT} } \right) + \varphi_{SWCNT} \frac{{\rho_{SWCNT} }}{{\rho_{f} }}, \\ \frac{{\rho_{hnf} }}{{\rho_{f} }} & = \left( {1 - \varphi_{SWCNT} - \varphi_{DWCNT} } \right) + \frac{{\varphi_{SWCNT} \rho_{SWCNT} + \varphi_{DWCNT} \rho_{DWCNT} }}{{\rho_{f} }}. \\ \end{aligned}$$

Mono/hybrid nanofluids' effective specific heat is articulated as:

13 $$\begin{aligned} \frac{{\left( {\rho C_{p} } \right)_{nf} }}{{\left( {\rho C_{p} } \right)_{f} }} & = \left( {1 - \varphi_{SWCNT} } \right) + \varphi_{SWCNT} \frac{{\left( {\rho C_{p} } \right)_{SWCNT} }}{{\left( {\rho C_{p} } \right)_{f} }}, \\ \frac{{\left( {\rho C_{p} } \right)_{hnf} }}{{\left( {\rho C_{p} } \right)_{f} }} & = \left( {1 - \varphi_{SWCNT} - \varphi_{DWCNT} } \right) + \frac{{\varphi_{SWCNT} \left( {\rho C_{p} } \right)_{SWCNT} + \varphi_{DWCNT} \left( {\rho C_{p} } \right)_{DWCNT} }}{{\left( {\rho C_{p} } \right)_{f} }}. \\ \end{aligned}$$

For effective thermal conductivity of the Mono/hybrid nanofluid Yamada-Ota model^43^ is considered and the estimates for $L$ and $R$ are taken as $10\mu m$ and $150nm$ respectively:

14 $$\begin{aligned} \frac{{k_{hnf} }}{{k_{nf} }} & = \frac{{1 + \frac{{k_{f} }}{{k_{DWCNT} }}\frac{L}{R}\varphi_{DWCNT}^{0.2} + \left( {1 - \frac{{k_{f} }}{{k_{DWCNT} }}} \right)\varphi_{DWCNT} \frac{L}{R}\varphi_{DWCNT}^{0.2} + 2\varphi_{DWCNT} \left( {\frac{{k_{DWCNT} }}{{k_{DWCNT} - k_{f} }}} \right)\ln \left( {\frac{{k_{DWCNT} + k_{f} }}{{2k_{DWCNT} }}} \right)}}{{1 - \varphi_{DWCNT} + 2\varphi_{DWCNT} \left( {\frac{{k_{f} }}{{k_{DWCNT} - k_{f} }}} \right)\ln \left( {\frac{{k_{DWCNT} + k_{f} }}{{2k_{f} }}} \right)}}, \\ \frac{{k_{nf} }}{{k_{f} }} & = \frac{{1 + \frac{{k_{f} }}{{k_{SWCNT} }}\frac{L}{R}\varphi_{SWCNT}^{0.2} + \left( {1 - \frac{{k_{f} }}{{k_{SWCNT} }}} \right)\varphi_{SWCNT} \frac{L}{R}\varphi_{SWCNT}^{0.2} + 2\varphi_{SWCNT} \left( {\frac{{k_{SWCNT} }}{{k_{SWCNT} - k_{f} }}} \right)\ln \left( {\frac{{k_{SWCNT} + k_{f} }}{{2k_{SWCNT} }}} \right)}}{{1 - \varphi_{SWCNT} + 2\varphi_{SWCNT} \left( {\frac{{k_{f} }}{{k_{SWCNT} - k_{f} }}} \right)\ln \left( {\frac{{k_{SWCNT} + k_{f} }}{{2k_{f} }}} \right)}}, \\ \end{aligned}$$

Mono/hybrid nanofluid effective electrical conductivity is devised as:

15 $$\begin{gathered} \frac{{\sigma_{hnf} }}{{\sigma_{nf} }} = \frac{{\sigma_{DWCNT} - 2\varphi_{DWCNT} \left( {\sigma_{f} - \sigma_{DWCNT} } \right) + 2\sigma_{f} }}{{\sigma_{DWCNT} + \varphi_{DWCNT} \left( {\sigma_{f} - \sigma_{DWCNT} } \right) + 2\sigma_{f} }}, \hfill \\ \frac{{\sigma_{nf} }}{{\sigma_{f} }} = \frac{{\sigma_{SWCNT} + 2\varphi_{SWCNT} \left( {\sigma_{f} - \sigma_{SWCNT} } \right) + 2\sigma_{f} }}{{\sigma_{SWCNT} + \varphi_{SWCNT} \left( {\sigma_{f} - \sigma_{SWCNT} } \right) + 2\sigma_{f} }}. \hfill \\ \end{gathered}$$

### Quantities of physical interest

The physical attributes known as surface drag coefficients and Nusselt number are formulated as:

16 $$C_{fx} = \frac{{2\tau_{zx} }}{{\rho_{f} u_{w}^{2} }},\,\,\,\,\,C_{fy} = \frac{{2\tau_{zy} }}{{\rho_{f} v_{w}^{2} }},\;\;\;\;Nu_{x} = \frac{{xq_{w} }}{{k_{f} \left( {T_{w} - T_{\infty } } \right)}},$$

where the shear stresses $\tau_{zx} ,\tau_{zy}$ and heat flux are computed as:

17 $$\tau_{zx} = \mu_{hnf} \left( {\frac{\partial u}{{\partial z}}} \right)_{z = 0} ,\,\,\,\tau_{zy} = \mu_{hnf} \left( {\frac{\partial v}{{\partial z}}} \right)_{z = 0} ,\;\;\;\;\left. \begin{gathered} PST\,\,\,\,\,q_{w} = - k_{hnf} \frac{\partial T}{{\partial z}} \hfill \\ PHF\,\,\,q_{w} = \frac{{\sqrt {\frac{a}{{v_{f} }}} \left( {T - T_{\infty } } \right)k_{hnf} }}{\Theta \left( \eta \right)} \hfill \\ \end{gathered} \right\}.$$

Using similarity variables (6), we get:

18 $$\begin{gathered} \sqrt {{\text{Re}}_{x} } C_{fx} = \frac{{\mu_{hnf} }}{{\mu_{f} }}f^{^{\prime\prime}} \left( 0 \right),\,\,\,\,\,\,\,\,\sqrt {{\text{Re}}_{y} } C_{fy} = \alpha^{\frac{3}{2}} \frac{{\mu_{hnf} }}{{\mu_{f} }}g^{^{\prime\prime}} \left( 0 \right),\,\,\,\,\,\,\,\, \hfill \\ \left( {PST} \right)\,\frac{{Nu_{x} }}{{\sqrt {{\text{Re}}_{x} } }} = - \frac{{k_{hnf} }}{{k_{f} }}\theta^{^{\prime}} \left( 0 \right),\,\,\,\,\left( {PHF} \right)\frac{{Nu_{x} }}{{\sqrt {{\text{Re}}_{x} } }} = \frac{{k_{hnf} }}{{k_{f} }}\frac{1}{\Theta \left( 0 \right)},\, \hfill \\ \end{gathered}$$

where ${\text{Re}}_{x} = \frac{{ax^{2} }}{{\nu_{f} }}$ and ${\text{Re}}_{y} = \frac{{ay^{2} }}{{\nu_{f} }},$ are the local Reynold numbers.

### Numerical analysis

**Figure 2:**
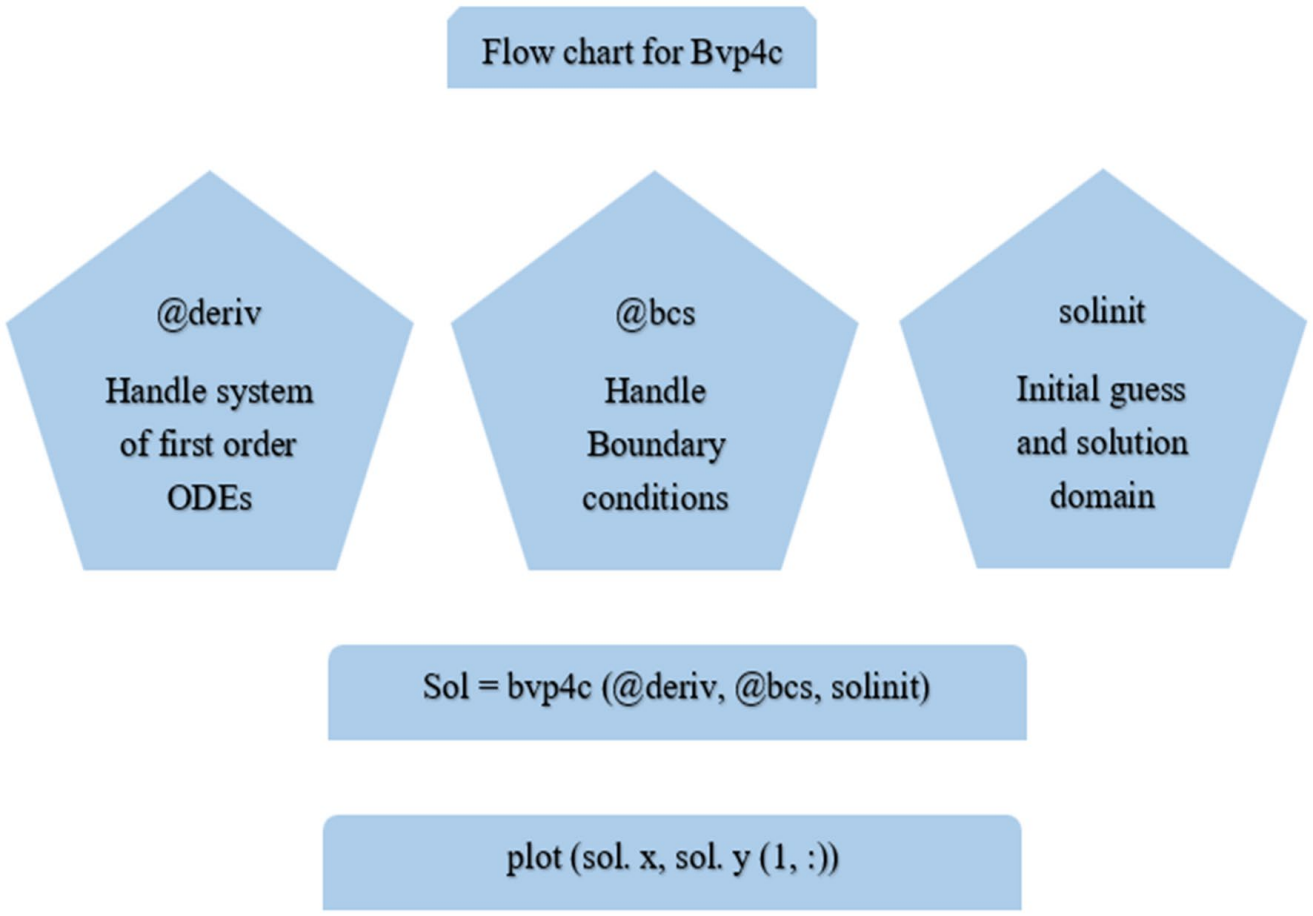
Flow chart of the bvp4c scheme.

Nanofluid flows are numerically computed by different techniques^44–57^. The “bvp4c” scheme, is used to compute the boundary value equations. The bvp4c technique is the finite difference approach. The modified ODEs (7)–(9) and their boundary constraints (10) are numerically investigated by employing the aforementioned method. Further, when the error involved reaches $10^{ - 6}$, the iterative procedure will be stopped. Moreover, Fig. 2 is added to elaborate on the numerical scheme used.

### Results analysis

**Figure 3:**
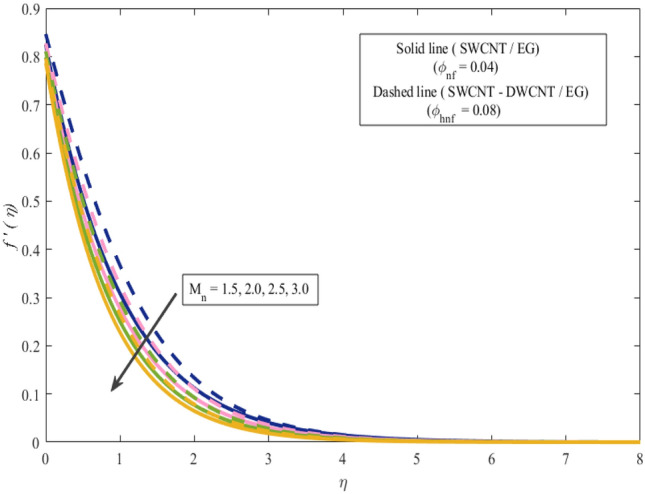
Upshot of magnetic parameter $M_{n}$ over $f^{^{\prime}} \left( \eta \right)$.

**Figure 4:**
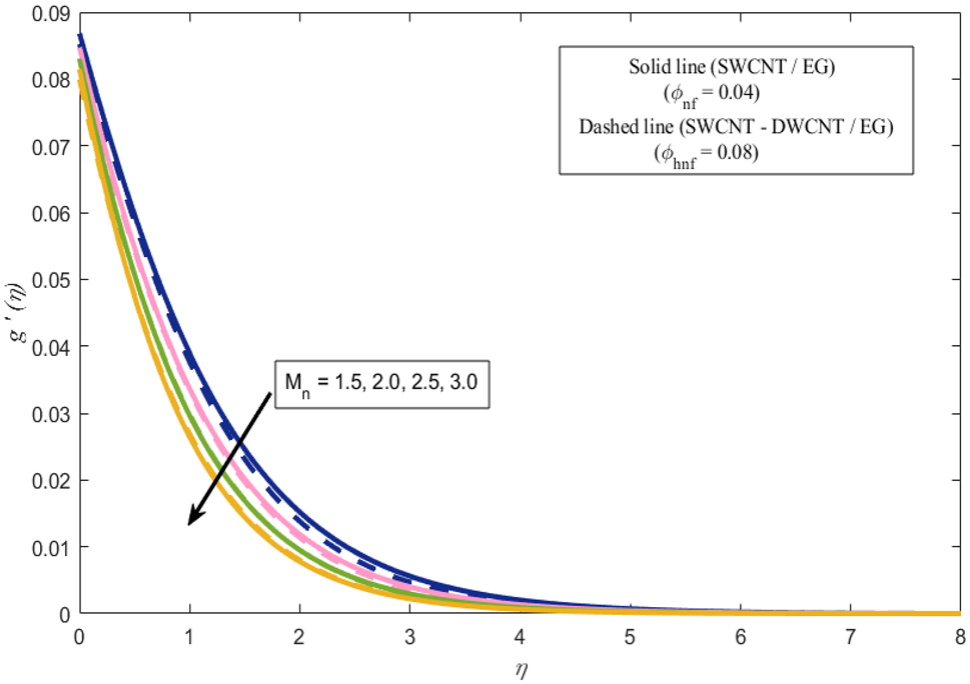
Upshot of magnetic parameter $M_{n}$ over $g^{^{\prime}} \left( \eta \right)$.

**Figure 5:**
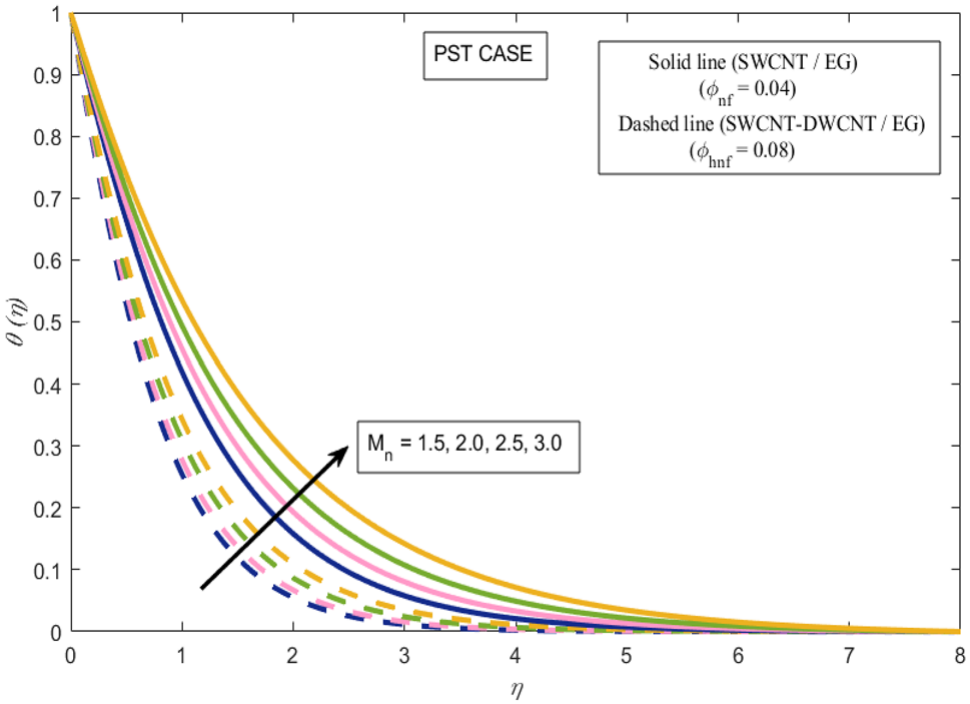
Upshot of magnetic parameter $M_{n}$ over $\theta \left( \eta \right)$.

**Figure 6:**
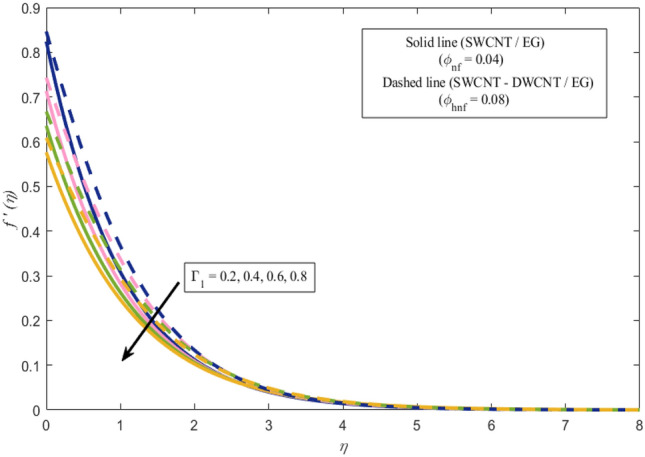
Upshot of velocity slip parameter $\Gamma_{1}$ over $f^{^{\prime}} \left( \eta \right)$.

**Figure 7:**
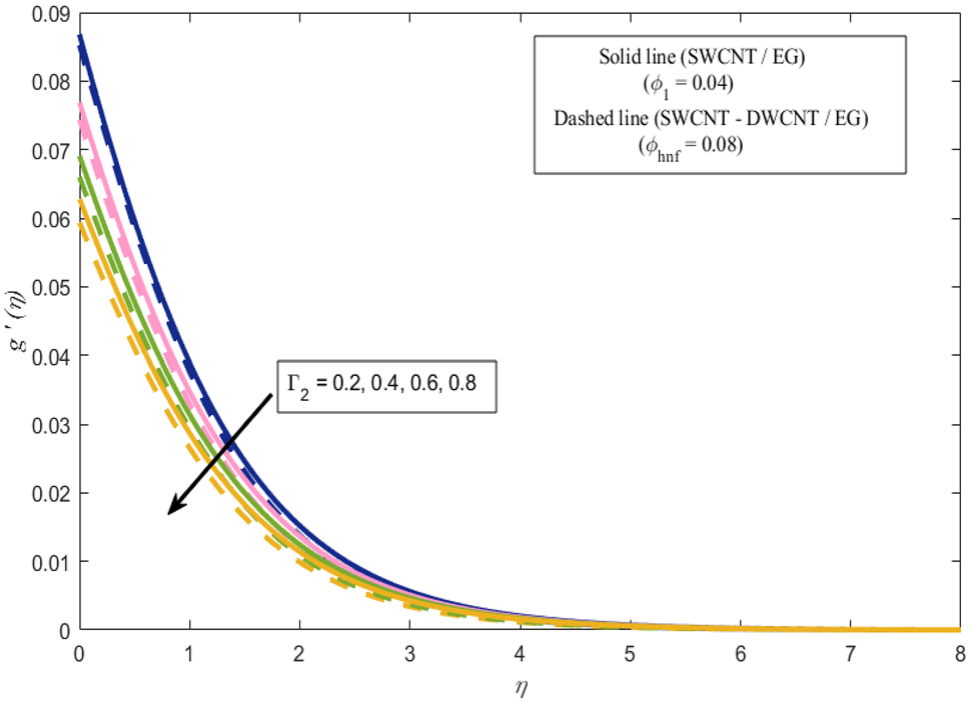
Upshot of velocity slip parameter $\Gamma_{2}$ over $g^{^{\prime}} \left( \eta \right)$.

**Figure 8:**
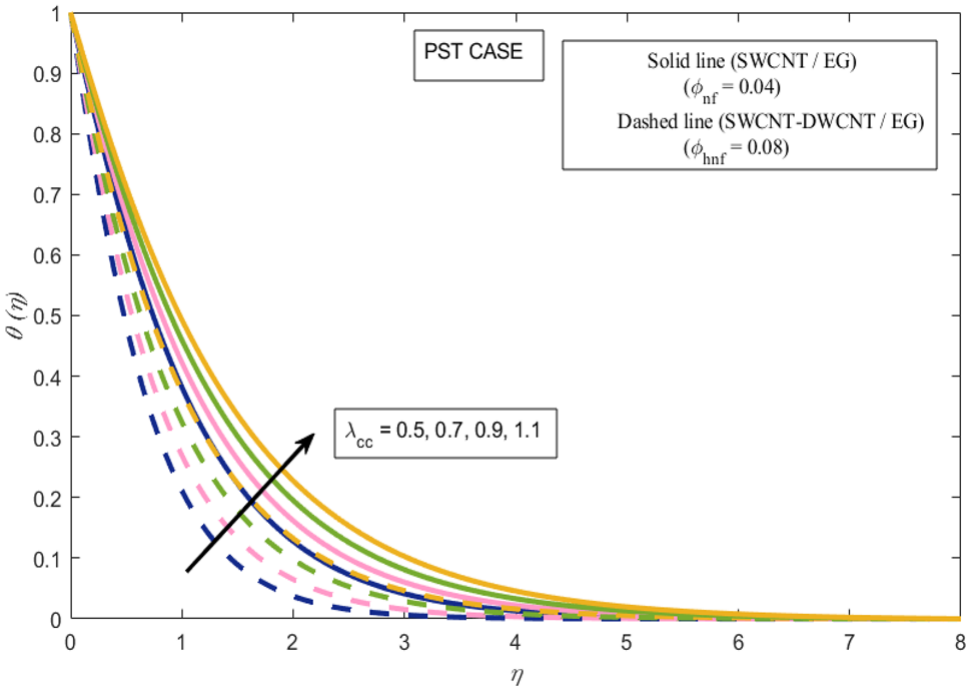
Upshot of thermal relaxation parameter $\lambda_{cc}$ over $\theta \left( \eta \right)$.

**Figure 9:**
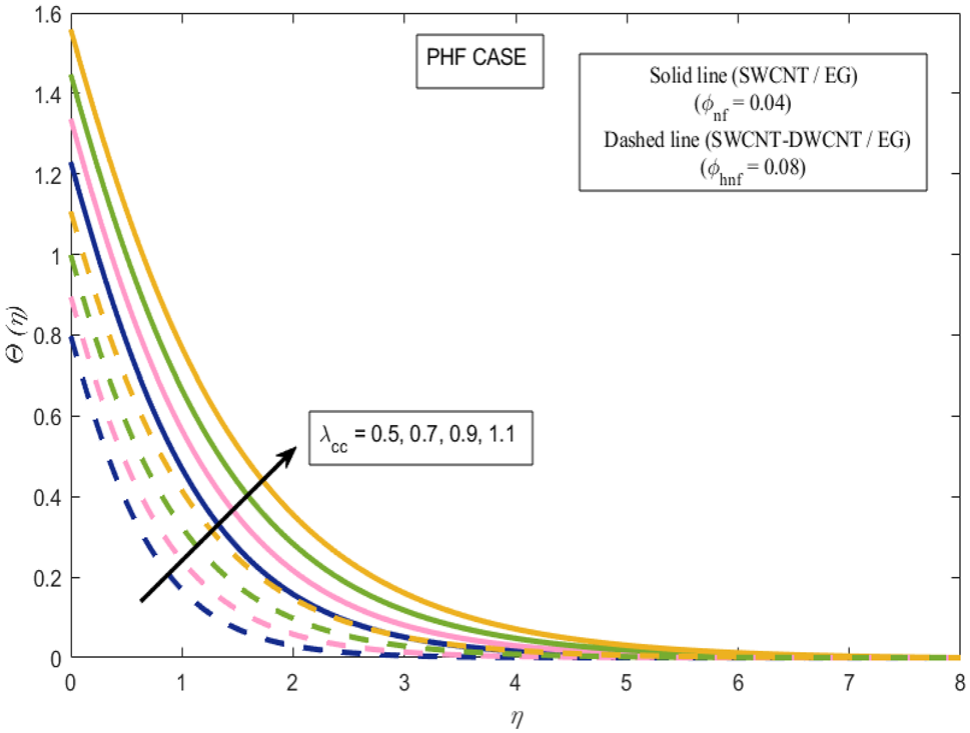
Upshot of thermal relaxation parameter $\lambda_{cc}$ over $\Theta \left( \eta \right)$.

**Figure 10:**
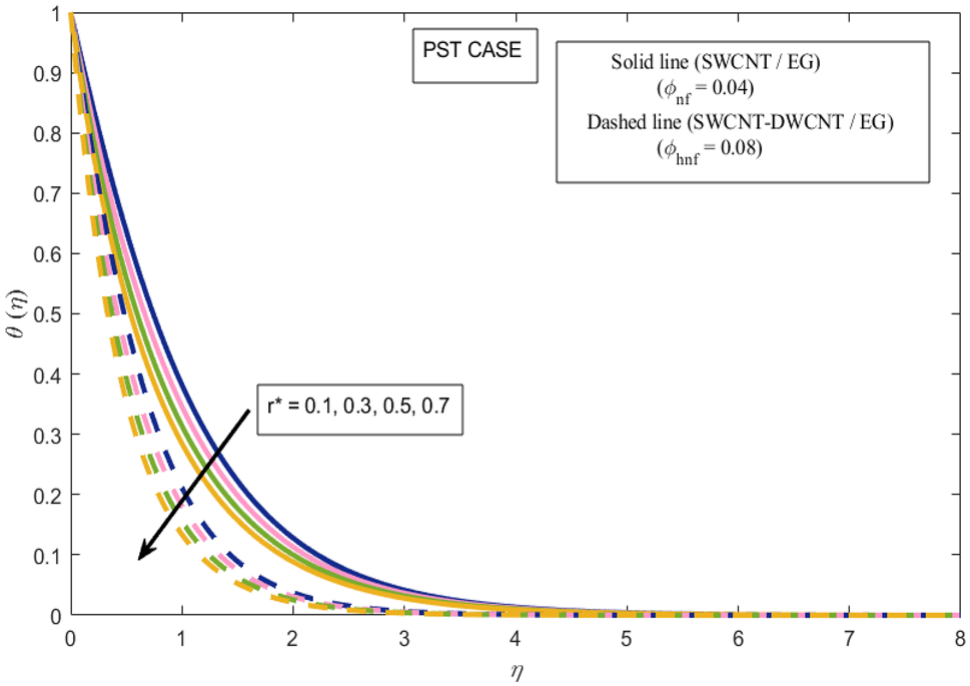
Upshot of power index $r^{ * }$ over $\theta \left( \eta \right)$.

**Figure 11:**
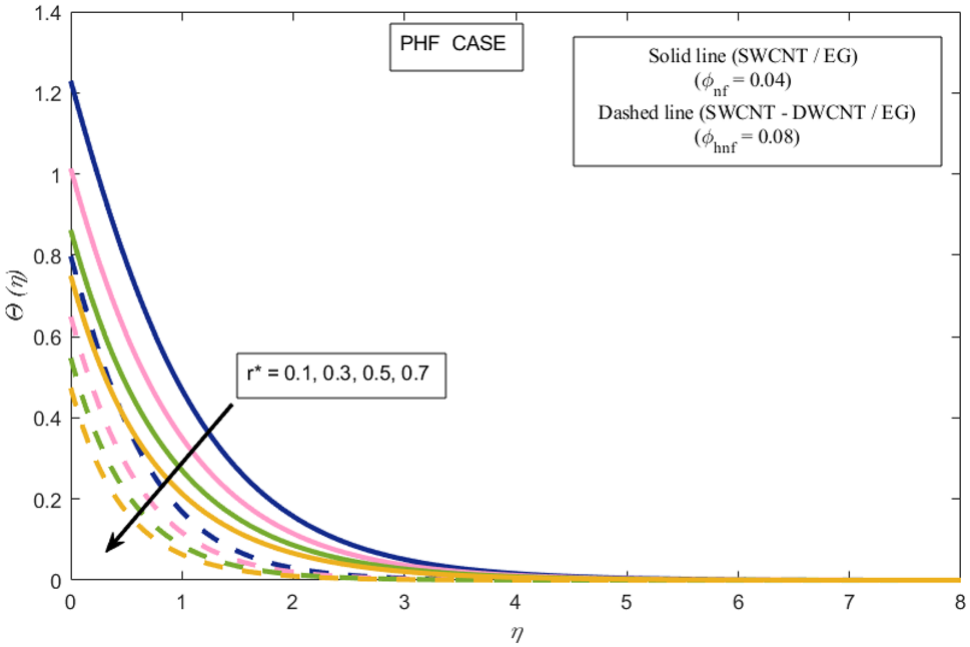
Upshot of power index $r^{ * }$ over $\Theta \left( \eta \right)$.

**Figure 12:**
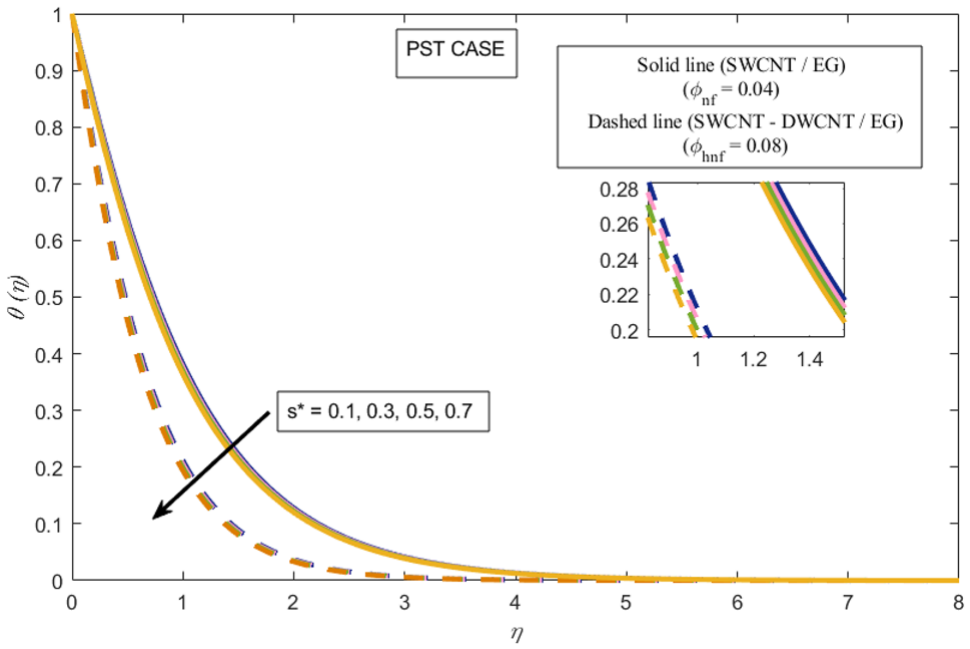
Upshot of power index $s^{ * }$ over $\theta \left( \eta \right)$.

**Figure 13:**
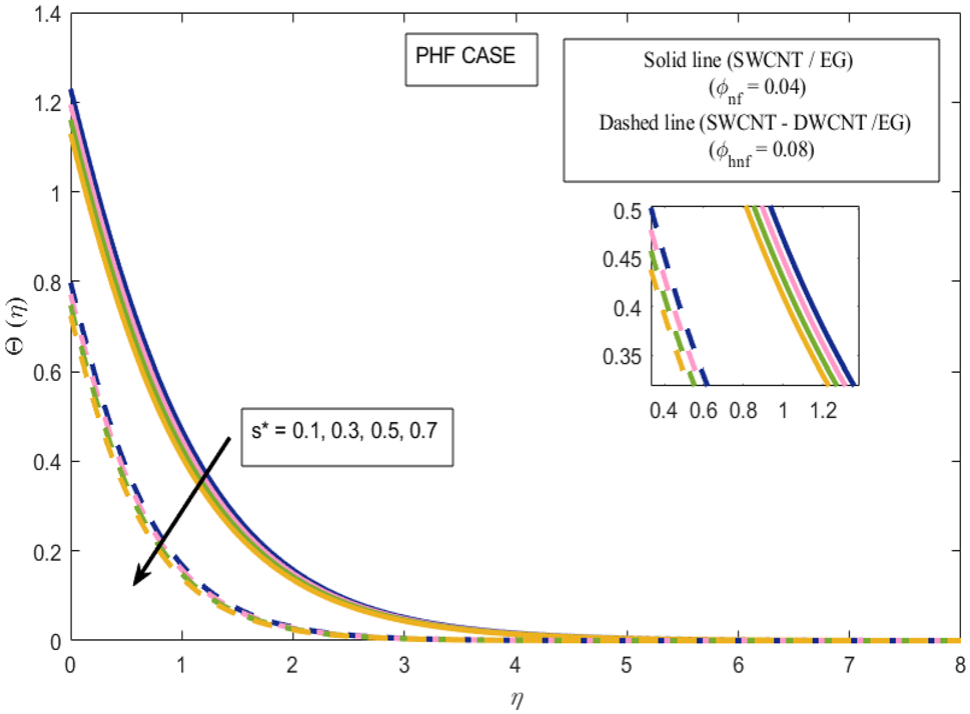
Upshot of power index $s^{ * }$ over $\Theta \left( \eta \right)$.

**Figure 14:**
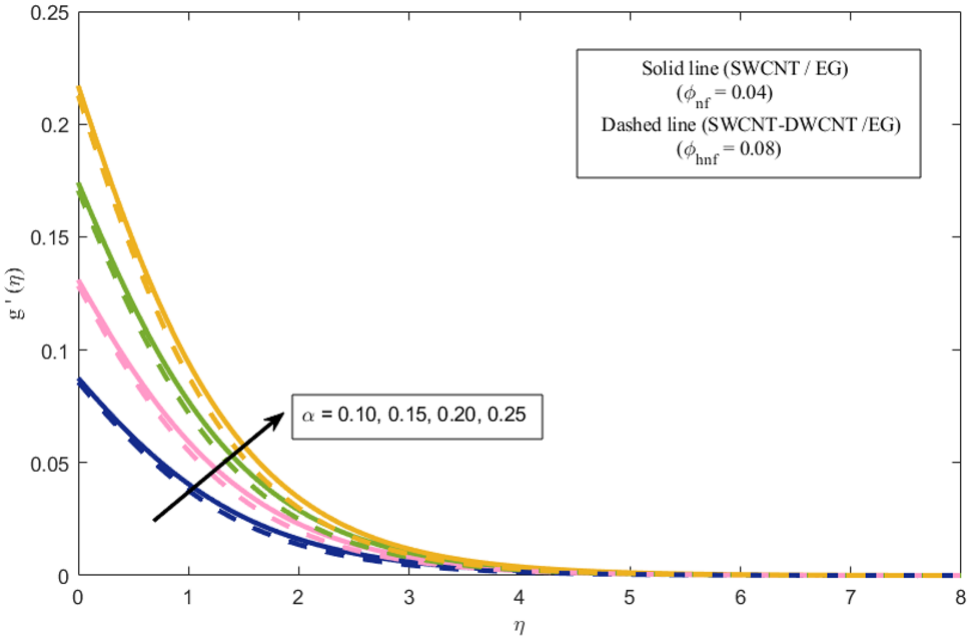
Upshot of stretching ratio parameter $\alpha$ over $g^{^{\prime}} \left( \eta \right)$.

This section presents a graphical analysis of numerous parameters versus velocity and temperature profiles with logical illustrations. In each figure, the comparison of mono and hybrid nanofluid is also provided. Furthermore, thermal profiles for both the prescribed surface temperatures are displayed separately. To depict the consequence of magnetic parameter $M_{n}$ against primary and secondary velocities, Figs. 3 and 4 are portrayed. It is noted that by upsurging the magnetic parameter $M_{n}$ both the primary $f^{^{\prime}} \left( \eta \right)$ and secondary velocities $g^{^{\prime}} \left( \eta \right)$ of the fluid dwindled. Physically, Lorentz's force resists the fluid motion that eventually lowers both velocities. This decreasing tendency is more pronounced for (SWCNTs/EG) than (SWCNTs-DWCNTs/EG). Further, Fig. 5 is plotted to visualize the consequence of magnetic parameter against the temperature profile $\theta \left( \eta \right)$. The fluid temperature is enhanced for rising numbers of magnetic parameter. Physically, more heat is generated due to the Lorentz force as the parameter $M_{n}$ rises, which eventually upsurges the temperature of the fluid. This enhancing trend is more significant for the (SWCNTs/EG) combination than for (SWCNTs-DWCNTs/EG) mixture. Figures 6 and 7 exhibit the graphical representation of the effect of directional dependent velocity slip parameters $\Gamma_{1}$ and $\Gamma_{2}$ against primary $f^{^{\prime}} \left( \eta \right)$ and secondary $g^{^{\prime}} \left( \eta \right)$ velocities. Both velocities declined against rising values of slip parameters. By increasing slip parameters, friction force may be generated, which in turn slows down flow velocity by allowing more fluid to slide over the sheet. Additionally, the decreasing trend is more obvious for (SWCNTs/EG) instead of (SWCNTs-DWCNTs/EG). Figures 8 and 9 are drawn to demonsrtate the consequences of the thermal relaxation parameter on fluid temperatures $\theta \left( \eta \right)$, $\Theta \left( \eta \right)$ for PST and PHF cases respectively. It is inferred that the temperature of the liquid escalates for mounting estimates of the thermal relaxation parameter $\lambda_{cc}$. For the PST and PHF cases, the escalating trend is more significant for (SWCNT/EG) than (SWCNT-DWCNT/EG). It is revealed that a great amount of $\lambda_{cc}$ produces faster boundary movement along $x$-direction that diminishes the stretching ratio parameter $\left( {\alpha = \frac{b}{a}} \right)$, that is why temperature is upsurging for the parameter $\lambda_{cc}$. The power indices $r^{ * }$ and $s^{ * }$ regulate the non-uniformity of the wall temperature for both the PST and PHF scenarios. Figures 10 and 11 explored the trend of the power index $r^{ * }$ on the temperatures $\theta \left( \eta \right)$, $\Theta \left( \eta \right)$ for both the PST and PHF cases respectively. It is described that the temperature distributions are significantly on the decline for higher estimations of $r^{ * }$. This is because of mounting estimates of the power index $r^{ * } ,$ the temperature difference increases, which subsequently enhances the heat transfer rate and reduces the temperature distributions. The declining trend is significant for (SWCNT-DWCNT/EG) than (SWCNT/EG). Figures 12 and 13 are illustrated to depict the influence of the power index $s^{ * }$ on both profiles $\theta \left( \eta \right)$, $\Theta \left( \eta \right).$ The figures indicate that for higher values of $s^{ * } ,$ the temperature profiles reduce for both the PST and PHF cases. Physically, for escalating estimations of the $s^{ * }$, the thermal gradient increases, which upsurges the heat transfer rate and results in the decline of the temperature profile. This diminishing trend is more obvious for (SWCNT-DWCNT/EG) than (SWCNT/EG). Figure 14 is added to describe the impact of the stretching ratio parameter $\alpha$ against the secondary profile. It is understood that the secondary profile $g^{^{\prime}} \left( \eta \right)$ upsurges for higher estimates of $\alpha$. This enhancement is due to the boundary condition $\alpha = \frac{b}{a}$, as stretching ratio increases the stretching rate $^{^{\prime}} b^{^{\prime}}$ escalates in the $y -$ direction which upsurges the velocity profile $g^{^{\prime}} \left( \eta \right)$ in that direction. The enhancing trend is more significant for hybrid nanofluid (SWCNT-DWCNT/EG) than mono nanofluid (SWCNT/EG).

Considering the importance of engineering quantities, Tables 2 and 3 are erected to determined the consequence of varied factors on the surface drag coefficients $C_{fx}$ and $C_{fy}$ along $x$, $y -$ directions for both mono-hybrid nanofluids. The tables illustrate that for mounting values of the slip parameter $\Gamma_{1}$, both the surface drag coefficients increase in the case of mono/hybrid nanofluids. For increasing estimates of stretching ratio $\alpha$ and magnetic $M_{n}$ parameters, the surface drag coefficients reduce for both mono and hybrid nanofluids. By increasing the particle volume fraction for mono nanofluid $\left( {\phi_{SWCNT} = 0.04,\,0.05,\,0.06,\,0.07} \right),$ the surface drag coefficients reduce. Moreover, to address the effect of particle volume fraction for hybrid nanofluid on surface drag coefficients, the value of $\phi_{SWCNT} = 0.04,$ is fixed and the particle volume fraction for hybrid nanofluid is taken as $\left( {\phi_{hnf} = \phi_{SWCNT} + \phi_{DWCNT} = 0.08,\,0.09,\,0.10,\,0.11} \right)$. Here, it is revealed that the skin friction coefficients reduce for greater values of particle volume fraction for hybrid nanofluid. Interestingly, surface drag coefficients are higher for (SWCNT/EG) as compared to (SWCNT-DWCNT/EG), and consequently, the use of the mono nanofluid will require a larger pumping power.

**Table 2:** Numerical results of surface drag coefficient $C_{fx}$ for mono/hybrid nanofluids.

$\Gamma_{1}$	$\alpha$	$M_{n}$	$\phi$	SWCNT/EG	SWCNT-DWCNT/EG
0.2	0.1	1.0	0.04	− 1.1289811	− 1.2012110
0.4				− 0.0031449	− 0.9449821
0.6				− 0.0031141	− 0.7829551
0.8				− 0.0030912	− 0.6702312
	0.15			− 1.1324579	− 1.2048811
	0.20			− 1.1357823	− 1.2085219
	0.25			− 1.1391167	− 1.2121120
		1.5		− 1.2350876	− 1.3125341
		2.0		− 1.3262912	− 1.4078572
		2.5		− 1.4064376	− 1.4913513
			0.05	− 1.1446170	− 1.2180416
			0.06	− 1.1604318	− 1.2351921
			0.07	− 1.1764436	− 1.2523878

**Table 3:** Numerical results of surface drag coefficient $C_{fy}$ for mono/hybrid nanofluids.

$\Gamma_{1}$	$\alpha$	$M_{n}$	$\phi$	SWCNT/EG	SWCNT-DWCNT/EG
0.2	0.1	1.0	0.04	− 0.0031894	− 0.0034182
0.4				− 0.0031449	− 0.0033678
0.6				− 0.0031141	− 0.0033335
0.8				− 0.0030912	− 0.0033083
	0.15			− 0.0088923	− 0.0095254
	0.20			− 0.0184607	− 0.0197652
	0.25			− 0.0326009	− 0.0348887
		1.5		− 0.0036008	− 0.0038478
		2.0		− 0.0039402	− 0.0042012
		2.5		− 0.0042304	− 0.0045026
			0.05	− 0.0032370	− 0.0034702
			0.06	− 0.0032853	− 0.0035229
			0.07	− 0.0033342	− 0.0035763

Further, the Nusselt number is computed for both thermal conditions, i.e., (PST) in Table 4 and, (PHF) in Table 5. It is demonstrated in Table 4 that, for higher values of power indices $r^{ * }$ and $s^{ * }$(which control the surface temperature), particle volume fractions $\left( {\phi_{nf} ,\phi_{hnf} } \right)$, magnetic parameter $M_{n} ,$ and stretching ratio parameter $\alpha ,$ the heat transfer rate on the wall enhances for all the parameters except the magnetic parameter. Hence, for the PST case, an increase in Nusselt number is substantial for (SWCNT-DWCNT/EG), in comparison to (SWCNT/EG). Table 5 depicts the similar behavior of the wall heat transfer rate against higher estimates of power indices $r^{ * }$ and $s^{ * }$ particle volume fractions $\left( {\phi_{nf} ,\phi_{hnf} } \right),$ magnetic parameter $M_{n} ,$ and stretching ratio parameter $\alpha .$ Moreover, for the prescribed surface heat flux more heat is transferred considering hybrid nanofluid than mono nanofluid. It may be inferred that in cooling processes, more heat transfer from the surface can be accomplished by employing a prescribed surface heat flux boundary condition than the prescribed surface temperature. To authenticate the current results with the findings from earlier publications, Table 6 is added. A good correlation is achieved.

**Table 4:** Numerical results of heat transfer rate $Nu_{x}$ for mono/hybrid nanofluids for PST case.

PST case						
$r^{ * }$	$s^{ * }$	$\phi$	$M_{n}$	$\alpha$	SWCNT/EG	SWCNT-DWCNT/EG
0.1	0.1	0.04	1.0	0.10	1.4581212	1.7494832
0.3					3.5971390	4.9967154
0.5					6.2380654	9.1008112
0.7					9.2302312	13.773766
	0.3				1.7247545	2.1298734
	0.5				2.0101153	2.5428411
	0.7				2.3140728	2.9886389
		0.05			1.6311250	2.0830566
		0.06			1.8043114	2.4370509
		0.07			1.9779795	2.8115562
			1.5		1.5220623	1.8229445
			2.0		1.5007100	1.8121377
			2.5		1.4346488	1.8075378
				0.15	1.4594190	1.7548756
				0.20	1.4622613	1.7620387
				0.25	1.4663184	1.7706120

**Table 5:** Numerical results of heat transfer rate $Nu_{x}$ for mono/hybrid nanofluids for PHF case.

PHF Case						
$r^{ * }$	$s^{ * }$	$\phi$	$M_{n}$	$\alpha$	SWCNT/EG	SWCNT-DWCNT/EG
0.1	0.1	0.04	1.0	0.10	1.9242261	6.9379993
0.3					2.3337743	8.5329832
0.5					2.7449899	10.107410
0.7					3.1577735	11.673049
	0.3				1.9809592	7.1732653
	0.5				2.0376425	7.4074462
	0.7				2.0942744	7.6405496
		0.05			2.0413092	8.5375267
		0.06			2.1480372	10.267358
		0.07			2.2461442	12.127011
			1.5		1.8747261	6.8764935
			2.0		1.8229236	6.7942819
			2.5		1.7709749	6.7000011
				0.15	1.9535796	7.0068883
				0.20	1.9802581	7.0673196
				0.25	2.0048247	7.1201435

**Table 6:** Comparison of outcomes with already existing work of Ramzan et al.^20^ for different estimates of $\alpha$ when $Mn = \Gamma_{1} = \Gamma_{2} = \Pr = r^{ * } = s^{ * } = \lambda_{cc} = \phi_{SWCNT} = \phi_{DWCNT} = 0$.

Stretching Parameter	Ramzan et al.^20^	Present
$\alpha$	$- g^{^{\prime\prime}} \left( 0 \right)$	
0	0	0
0.1	0.073015	0.073014
0.2	0.158223	0.158222
0.3	0.254345	0.254344
0.4	0.360590	0.360589
0.5	0.476291	0.476290

## Conclusion

We have scrutinized the comparative analysis of mono and hybrid MHD nanofluid flows due to a bidirectional extending surface with modified Fourier law and anisotropic slip constraints. The process of heat transfer is analyzed with two thermal conditions that are applied to the surface: prescribe surface temperature and prescribe heat flux. In this work, two distinct nanoparticles, SWCNT and DWCNT, are inserted in the base fluid ethylene glycol. The bvp4c numerical scheme is applied to handle the boundary layer equations by first converting them to an ODE system of order one. The following are the most important observations from the current findings:

- By enhancing the magnetic parameter both the primary and secondary profiles reduce. Whereas the temperature of the fluid enhances. This enhancement is more obvious for mono nanofluid (SWCNT/EG) instead of hybrid nanofluid (SWCNT-DWCNT/EG).
- Secondary and primary velocity distributions for both the nanofluids are reduced by enhancing the directional dependent slip coefficients.
- For PST and PHF scenario, the temperature distributions upsurges for large estimations of the thermal relaxation parameter. Compared to hybrid nanofluid (SWCNT-DWCNT/EG), this trend of enhancement is more obvious for mono nanofluid (SWCNT/EG).
- Under the PST and PHF scenarios, the estimations of the surface temperature with power indices has a significant consequence on the thermal boundary layer. The reduced boundary layer is noted more for the stretching of the sheet in $y -$ direction instead of $x -$ direction.
- For higher estimates of the stretching ratio factors the secondary velocity distributions enhances.
- Heat transfer rate is escalated for hybrid nanoliquid (SWCNT-DWCNT/EG) instead of mono nanofluid (SWCNT/EG), whereas surface drag coefficients are higher for mono nanofluid instead of hybrid nanofluid.
- Ethylene glycol (EG) is preferred due to its superior heat transfer and anti-freezing properties. It follows that a hybrid nanofluid (SWCNT-DWCNT/EG) with a heat flux boundary condition will be preferred because nano-coolant is more suited for radiators and other cooling systems than a mono nanofluid (SWCNT/EG).

### Applications of the presented model

In this study, hybrid nanofluid is used due to its advanced heat transfer properties as compared to mono nanofluid. Ethylene glycol (EG) is employed as a base liquid due to its superior heat transfer and anti-freezing properties. Further, Prescribed surface temperature and heat flux boundary constraints are utilized because, in the steelmaking industry, it is important to control the temperature and heating rates to achieve a good quality of the end products.

### Future scope

This research can be extended in the following directions:

- The hybrid nanofluid flow can be considered in the spongy medium.
- The model can be evaluated by adopting a ternary hybrid nanofluid flow.
- Xue thermal conductivity model can be used for carbon nanotubes.
- Boundary conditions can be replaced by thermal jump and melting heat transfer.

### Commentaries

The commentaries point out two potential observations that may be unexpected or surprising based on prior knowledge or assumptions:

- *Slip coefficients value can be higher than one*: The slip coefficient is a dimensionless quantity that connects the shear stress at the contact to the slip velocity between two fluid layers. A slip coefficient value larger than one shows that the slip velocity exceeds the velocity differential between the two fluids, which traditional models do not predict. The comments indicate that such big slip coefficient values are possible under certain conditions, challenging previous assumptions and opening up new research avenues.
- *Magnetic field strength values can be less than zero*: Magnetic field strength is a measure of the intensity of a magnetic field. Because magnetic fields are commonly assumed to be positive vector quantities, a negative magnetic field strength value may appear counterintuitive or impossible. The viewpoints demonstrate that such negative values may exist in some settings, calling into question previous assumptions and needing new theoretical or experimental approaches to comprehend.

## Data Availability

All data generated or analyzed during this study are included in this published article.

## Abbreviations

$$r^{ * } ,s^{ * }$$: Power indices
$$u_{w} ,v_{w}$$: Stretching velocities $$\left( {{\text{m}}/{\text{s}}} \right)$$
$\beta_{o}$: Magnetic field strength
$$\rho$$: Density $$\left( {{\text{kg}}/{\text{m}}^{3} } \right)$$
$\varphi$: Particle volume fraction
$T_{\infty }$: Temperature at ambient $\left( {\text{K}} \right)$
$$T_{0} ,T_{1}$$: Reference temperatures $$\left( {\text{K}} \right)$$
$$\nu$$: Kinematic viscosity $$\left( {{\text{m}}^{2} /{\text{s}}} \right)$$
$$x,y,z$$: Dimensionless coordinates $$\left( {\text{m}} \right)$$
$$\sigma$$: Electrical conductivity $$\left( {{\text{Sm}}^{ - 1} } \right)$$
$$T_{w}$$: Wall temperature $$\left( {\text{K}} \right)$$
$$\gamma_{1} ,\gamma_{2}$$: Slip coefficients
$a,b$: Lateral stretching rates $\left( {{\text{s}}^{ - 1} } \right)$
$\theta \left( \eta \right)$: Temperature for PST case $\left( {\text{K}} \right)$
$${\text{Re}}$$: Reynold number
$$f\left( \eta \right)$$: Dimensionless velocity
$u,v,w$: Components of velocity $\left( {{\text{m}}/{\text{s}}} \right)$
$$q_{w}$$: Heat flux
$\lambda_{cc}$: Thermal relaxation parameter
$L,R$: Length and radius of nanotube $\left( {\text{m}} \right)$
$$M_{n}$$: Magnetic parameter
$$k$$: Thermal conductivity $$\left( {{\text{W}}/{\text{mK}}} \right)$$
$$u{}_{w},v_{w}$$: Stretching velocities $$\left( {{\text{m}}/{\text{s}}} \right)$$
$\lambda$: Thermal relaxation constant
$$\alpha$$: Stretching ratio
$$\mu$$: Dynamic viscosity $$\left( {{\text{kg}}/{\text{ms}}} \right)$$
$\left( {C_{p} } \right)$: Specific heat capacity $\left( {{\text{J}}\,{\text{kg}}\,{\text{K}}^{ - 1} } \right)$
$$\Gamma_{1} ,\Gamma_{2}$$: Slip parameters
$$\Pr$$: Prandtl number
$\Theta \left( \eta \right)$: Temperature for PHF case $\left( {\text{K}} \right)$
$$\tau_{zx} ,\tau_{zy}$$: Shear stresses
$$g\left( \eta \right)$$: Dimensionless velocity

## Subscripts

$$nf$$: Nanofluid
$$f$$: Working fluid
$$hnf$$: Hybrid nanofluid
$\infty$: Conditions at ambient
